# ‘It does fill me with a bit of unease’: a qualitative study of the acceptability, facilitators and barriers to reducing the frequency of screening for asymptomatic sexually transmitted infections among gay, bisexual and other men who have sex with men

**DOI:** 10.1136/sextrans-2025-056556

**Published:** 2025-07-15

**Authors:** William Berners-Lee, Melissa Cabecinha, James Bell, Dawn Phillips, Tom Witney, Caisey V Pulford, Fabiana Lorencatto, Helen Fifer, Kirsty Foster, Hamish Mohammed, Katy Sinka, Deborah Williamson, Greta Rait, Kate Folkard, John Saunders

**Affiliations:** 1 Blood Safety, Hepatitis, Sexually Transmitted Infections (STI) and HIV Division, UK Health Security Agency—Colindale, London, UK; 2 The National Institute for Health Research Health Protection Research Unit in Blood Borne and Sexually Transmitted Infections at University College London in Partnership with the UK Health Security Agency, London, UK; 3 Research Department for Primary Care and Population Health, UCL, London, UK; 4 Department of Infection and Population Health, UCL, London, UK; 5 Centre for Behaviour Change, UCL, London, UK; 6 Institute of Epidemiology and Health, University College London, London, UK

**Keywords:** SEXUAL HEALTH, NEISSERIA GONORRHOEAE, Chalmydia Trachomatis, Diagnostic Screening Programs, QUALITATIVE RESEARCH

## Abstract

**Abstract:**

**Objectives:**

The study aimed to explore the acceptability of reducing the frequency of asymptomatic *Chlamydia trachomatis* (Ct) and *Neisseria gonorrhoeae* (Ng) screening among gay, bisexual, and other men who have sex with men (GBMSM)(Although the term GBMSM is used for convenience, the study also includes nonbinary people who were assigned male at birth who have sex with men.). Additionally, it sought to identify barriers and facilitators to implementing such changes and to develop potential interventions that could support a shift in current screening guidelines.

**Methods:**

This qualitative study explored stakeholder perspectives on reducing screening frequency and identified potential interventions that could support future guideline changes of this kind. Semistructured interviews were conducted with 22 GBMSM and 8 professional stakeholders. Data were thematically analysed using the Capabilty, Opportunity, Motivation - Behaviour (COM-B) and Theoretical Domains Framework (TDF). TDF domains were mapped to behaviour change techniques to inform intervention development. Candidate interventions were refined based on acceptability, practicability, effectiveness, affordability, side effects, equity.

**Results:**

Overall, GBMSM stakeholder responses to discontinuing asymptomatic Ng and Ct screening tended to be negative, while professional stakeholder opinions were mixed. Reducing the recommended screening frequency to 6 monthly was generally more acceptable to both groups. Barriers and facilitators to guideline changes included issues of knowledge and trust, social influence and identity, context and resources, concerns about consequences and emotional responses and habit. Ten candidate interventions were suggested. These involve providing information, social support, behavioural substitutions and feedback as well as facilitating discussions to resolve concerns.

**Conclusion:**

Any reduction in the recommended frequency of asymptomatic screening will encounter a range of interrelated barriers, including knowledge gaps, social influences and emotional factors. We identified evidence-based interventions that could improve acceptance and minimise unintended consequences. Future research should incorporate stakeholder workshops to refine these strategies.

**WHAT IS ALREADY KNOWN ON THIS TOPIC**

- The effectiveness of regular asymptomatic *Chlamydia trachomatis* (Ct) and *Neisseria gonorrhoeae* (Ng) screening of gay, bisexual and other men who have sex with men (GBMSM) for reducing harm has been called into question. There are also concerns that the practice may be contributing to rising antimicrobial resistance.

**WHAT THIS STUDY ADDS**

- This study highlights key barriers and facilitators to professional and GBMSM stakeholders accepting reduced asymptomatic Ng and Ct screening. Using behavioural science techniques, barriers have been mapped onto effective behaviour change techniques and candidate interventions to support acceptance of the guideline change.

**HOW THIS STUDY MIGHT AFFECT RESEARCH, PRACTICE OR POLICY**

- The shortlist of candidate interventions provides the first step in the codesign of effective public health interventions and should be followed with stakeholder engagement. This study contributes to a growing literature on sexual health attitudes among GBMSM to inform future public health interventions.

## Introduction

*Chlamydia trachomatis* (Ct) and *Neisseria gonorrhoeae* (Ng) are the most commonly diagnosed bacterial sexually transmitted infections (STIs) in England. In 2023, there were 194 970 Ct and 85 223 Ng diagnoses, with 21.5% occurring in gay, bisexual and other men who have sex with men (GBMSM).^1^

Three-monthly screening for Ct and Ng is recommended for GBMSM at higher STI risk,^2^ but this policy has been questioned for several reasons.^3^ First, screening has not reduced Ct or Ng prevalence.^4 5^ Second, most Ng and Ct infections in people assigned male at birth are asymptomatic—estimated at 70% for all chlamydia infections and up to 90% for rectal and pharyngeal infections.^6^ Finally, increased screening leads to higher antibiotic use, contributing to antimicrobial resistance (AMR).^7^,^9^

There is little evidence that 3 monthly screening reduces Ct or Ng prevalence more than annual screening, possibly because most infections are detected after the acute stage, when transmissibility is lower.^4 7^ Screening and treating asymptomatic cases may also accelerate AMR among key pathogens.^8^,^12^ The UK has recently seen rapid increases in Ng strains resistant to tetracycline and cefixime.^9^ More concerningly, ceftriaxone—the last first-line monotherapy for gonorrhoea—has shown resistance, with reported treatment failures for pharyngeal gonorrhoea using both ceftriaxone monotherapy and dual therapies (ceftriaxone plus azithromycin or doxycycline) in multiple countries.^10^

Given these concerns, the current test-and-treat strategy may be an inappropriate use of resources and could be causing more harm than benefit. There have been calls to reduce or stop asymptomatic Ng and Ct screening.^3^

However, the acceptability of reduced screening among GBMSM, healthcare providers and policymakers remains unclear and has been identified as a priority research question by international experts.^3^ This study aimed to (1) use a behavioural science perspective to qualitatively explore the acceptability, barriers and facilitators of reducing asymptomatic Ct and Ng screening frequency among GBMSM and (2) develop a shortlist of potential interventions to support any future reduction in screening.

## Methods

### Participants

We conducted qualitative interviews with two groups of participants: (1) cis or transgender GBMSM and non-binary people assigned male at birth reporting sex with men, aged 16 years and resident in the UK (GBMSM stakeholders) and (2) professional stakeholders working in clinical, policy, commissioning or community roles relating to sexual health. Participants were recruited through several channels, including adverts distributed by community organisation partners, the ‘Call for Participants’ website, and professional networks (for professional stakeholders only). Purposive sampling was used to ensure a range of professional roles, demographics, geographical location and experience of STI screening. Data collection continued until there was no emergence of new information from the interviews.^11 12^

### Procedure

Semistructured interviews (n=22 GBMSM; n=8 professional stakeholders) were conducted by a scientist (*WBL*, male)*,* a public health practitioner (*DP,* female), and an epidemiologist (*JB*, male) between 10 April 2024 and 9 August 2024. All interviewers had undertaken relevant qualitative research training and were professionally interested in sexual health. The interviewers had existing professional relationships with some professional stakeholders from previous projects. Topic guides were piloted and iteratively refined before data collection (see online supplemental appendices1 and 2 for topic guides). Written informed consent was obtained from all participants before the interview, which was confirmed verbally at the time of the interview. Interviews lasted an average of 60 min. All interviews were conducted via Microsoft Teams, with only the interviewee and research team members present. At the end of the interview, all participants were offered a £30 shopping voucher and provided with information about sexual health resources.

All interviews were audio-recorded and transcribed verbatim using Microsoft Teams’ automatic transcription, checked for accuracy and fully anonymised.

### Analysis

Transcripts were coded using NVivo V.14 by at least one researcher. Initially, all team members coded the same three transcripts to ensure consistency, meeting regularly to resolve uncertainties. The transcripts were initially coded within the six Capability, Opportunity, Motivation - Behaviour (COM-B) domains, through deductive thematic analysis.^13^ Themes were then coded according to the more granular Theoretical Domains Framework (TDF),^14^ before being refined by the wider team.

The COM-B model was chosen as an analytical framework for its broad applicability to drivers and barriers to behaviour change and ease of use in qualitative interviews. It posits that behaviour change requires capability, opportunity and motivation: psychological capability (knowledge and skills), physical capability (strength or stamina), physical opportunity (resources), social opportunity (social norms and cues), reflective motivation (beliefs and goals) and automatic motivation (emotions and habits).^15^

TDF domains identified as barriers to the implementation of reduced asymptomatic Ng and Ct screening were mapped to behaviour change techniques (BCTs).^16 17^ For each TDF domain, mapping tools were employed to identify evidence-based BCTs suitable for addressing the barriers and facilitators associated with that domain.^17^ Following this mapping, a range of candidate interventions was assembled. These were reviewed by the multidisciplinary team using the acceptability, practicability, effectiveness, affordability, side effects and equity (APEASE) criteria^17^ to produce a shortlist.

## Results

Twenty-two GBMSM stakeholders and eight professional stakeholders participated in the interview study. The characteristics of the GBMSM interviewees are reported in table 1. Professional stakeholders included sexual health service commissioners, genitourinary medicine consultants and representatives from community organisations.

**Table 1:** Characteristics of GBMSM stakeholders

Characteristics		GBMSM stakeholders, N=22 (%)
Age group	16–24	5 (22.7)
	25–34	9 (40.9)
	34–44	2 (9.1)
	45–54	4 (18.2)
	55–64	0 (0.0)
	65+	2 (9.1)
Gender	Cisgender male	18 (81.8)
	Transgender male	2 (9.1)
	Non-binary AMAB	2 (9.1)
Sexual behaviour	Men/AMAB only	15 (68.20
	Men and women	7 (31.8)
Ethnicity	White	13 (59.0)
	Black	2 (9.1)
	Asian	5 (22.7)
	Mixed	2 (9.1)
Number of STI screens in the last year	0	4 (18.2)
	1	1 (4.5)
	2	5 (22.7)
	3	5 (22.7)
	4	3 (13.6)
	5 or more	4 (18.2)
STI diagnosis in the last year	Yes	9 (40.9)
	No	13 (59.0)
HIV status	Negative	14 (63.6)
	Positive	4 (18.2)
	Never tested/not stated	4 (18.2)
Mental or physical health condition	Yes	8 (36.4)
	No	13 (59.0)
	Not specified	1 (4.5)
Born in the UK	Yes	18 (81.8)
	No	3 (13.6)
	Not specified	1 (4.5)
Region	London	6 (27.3)
	South-East	2 (9.1)
	South-West	0 (0.0)
	East-Midlands	2 (9.1)
	West-Midlands	2 (9.1)
	North-East	1 (4.5)
	North-West	6 (27.3)
	East of England	0 (0.0)
	Yorkshire and the Humber	3 (13.6)
Financial situation	Can’t cover personal expenses	3 (13.6)
	Struggles to cover personal expenses	2 (9.1)
	Managing to cover personal expenses	7 (31.8)
	Comfortably covers personal expenses	7 (31.8)
	Very comfortable financially	3 (13.6)

AMAB - Asigned Male at Birth

### Overall sentiment to proposed guideline changes

GBMSM stakeholder responses to discontinuing asymptomatic Ng and Ct screening were mostly negative, while professional stakeholder opinions were mixed. For both groups, reducing the recommended screening frequency from 3 monthly to 6 monthly was generally more acceptable than removing the recommendation entirely or reducing screening to every 12 months.

Themes within GBMSM and professional stakeholder responses are summarised below. Subthemes are summarised within broader themes. Illustrative quotes for each subtheme are provided in table 2. A mapping of barriers and facilitators to COM-B and TDF domains is provided in online supplemental appendix 4.

**Table 2:** Barriers to reducing the frequency of asymptomatic screening for sexually transmitted infections alongside illustrative quotes and suggested interventions

Barrier(s)	Illustrative quote(s)	Selected behaviour change technique(s)	Intervention target group(s)	Suggested intervention(s)
**Theme: Knowledge, trust and goals**				
Confidence and trust in the rationale behind reducing the frequency of asymptomatic screening	‘*From what you've explained now and what I've seen, I think it’s good enough reasons medically’*—GBMSM Stakeholder 71 ‘*I don't feel confident … that … only symptomatic infections … cause consequences?’*—Professional stakeholder 605	Information about health consequences Information about social and environmental consequences Salience of consequences	Professional and GBMSM stakeholders	**Intervention A** Providing information on: (1) the natural history of Ng and Ct, including a quantification of the risks associated with untreated infections, (2) the consequences of AMR for individuals and the community, and its association with STI screening.
		Goal setting (outcome)	GBMSM stakeholders GBMSM community organisations	**Intervention B** Sexual health professionals could facilitate discussions to establish shared goals. Goals could include reducing the health burden on GBMSM or ensuring that unnecessary testing and treatment is limited. These discussions could take place in one-to-one sexual health consulations as well as in larger facilitated sessions with GBMSM community organisations.
Priority of the goal of reducing unnecessary antibiotic use	‘*I don't feel like that the risk [from AMR] has been sort of quantified’*—Professional stakeholder 72	Goal setting (outcome) Review of outcomes goal	Sexual health service professionals	**Intervention C** Set reduction targets for antibiotic prescriptions following asymptomatic Ct and Ng diagnoses at sexual health services. Importantly treatments for symptomatic STIs and any syphilis infection should be excluded from these targets to avoid negative unintended consequences. Antibiotic prescription numbers could be reviewed and goals reevaluated at regular meetings with service providers.
**Theme: Concern about consequences**				
Concern about the personal sexual health impact … Concern about the community sexual health impact	‘*you could be doing immeasurable damage to your own body without realising it’*—GBMSM Stakeholder 699 … ‘*All it’s going to do is increase the number of people actually with STIs to a record level’* Participant 699 ‘*they may then transmit [infections] to partners, which might cause problems between relationships’*—Professional stakeholder 167			**See intervention A**
Concern about the structural sexual health impact … Concerns about the impact on attitudes towards Ng, Ct and other STIs	‘*You're not getting them in for regular sort of behavior change messaging, talking about partners and following up’*—Professional stakeholder 910 ‘*Less testing would, I guess, mean less strain on the system, which, as I've alluded to, feels quite strained when you're trying to get [tested*)’—GBMSM Stakeholder 311 … ‘*I think yeah, [screening less frequently] would make me less fearful of [STIs*)’—GBMSM Stakeholder 311 ‘*it’s a fine line between saying … only [test] if you're symptomatic whilst not losing the importance of syphilis, hepatitis, and HIV’*—Professional stakeholder 605	Information about social and environmental consequences Information about emotional consequences Pros and cons	Professional and GBMSM stakeholders	**Intervention D** Through facilitated discussions with both GBMSM and professional stakeholders a pros and cons list could be compiled. This could be presented to GBMSM and sexual health professionals, providing reassurance that the change has been codeveloped and that potential unintended consequences have been considered.
**Theme: Social influences and identity**				
Identity as a member of a GBMSM community	‘*If you kind of like going around without having been tested, it is not doing your your bit for the community at large’*—GBMSM Stakeholder 161	Information about others approval Social support (unspecified)	GBMSM stakeholders	**Intervention E** Provide testimonials from GBMSM who support reduced frequency of asymptomatic STI screening. Engage with GBMSM community organisations to encourage peer support for following new screening guidelines. This could be delivered in an online format (eg, GBMSM sharing personal screening practices with one another on social media groups).
		Credible source Behaviour substitution	GBMSM stakeholders	**Intervention F** To protect the social identity of GBMSM, new guidelines should be communicated by a credible source. In interviews, GBMSM stakeholders suggested that this could mean community organisations, GBMSM social media influencers, or healthcare professionals. Behaviour substitution could involve replacing asymptomatic screening with checking one’s body for symptoms or ensuring continued screening for other conditions, such as HIV and syphilis.
Identity as a sexual health service professional	‘*We've spent four decades telling gay men to test every three months for everything and [now we are] changing the message*.’—Professional stakeholder 72	Credible source Behaviour substitution	Sexual health service professionals	**Intervention G** New guidelines should also be communicated to sexual health professionals by a credible source, to protect their sense of identity. BASHH could fill this role. For professional stakeholders, behaviour substitution could involve replacing asymptomatic screening with providing advice on other aspects of sexual health.
**Theme: Context and resources**				
Burden on GBMSM from screening	*‘That obviously saves travel time to appointments or that time out of work, etcetera’*—GBMSM Stakeholder 794 ‘*if you’re a bottom, if you're having anal sex… you're not gonna get discharge in the penis … and because you’re total bottom, you're not going to know’*—GBMSM Stakeholder 699	Instruction on how to perform behaviour	GBMSM stakeholders	**Intervention H** Guidance on how to check for symptoms of Ng or Ct could be provided in a range of formats. Formats could be online (eg, information on dating apps or social media) or offline (eg, physical adverts and outreach workers).
**Theme: Emotional responses and habit**				
Anxiety about infrequent screening Sense of neglect regarding GBMSM sexual health	‘*in the short term this might have some impacts on overall well being of an individual from a mental health standpoint of view’*—GBMSM Stakeholder 957 ‘*I'd probably question [whether this is due to] a lack funding for clinics?’*—GBMSM stakeholder 794	Reduce negative emotions	GBMSM stakeholders	**Intervention I** To reduce negative emotions among GBMSM, empathy-focused communication and psychoeducation could be used. Empathy-focused communication training could be provided to sexual health professionals to ensure that anxiety and feelings of guilt related to contracting and transmitting STIs are addressed in consultations with GBMSM. The interventions designed to address knowledge and beliefs about consequences, could also serve as psychoeducation.
Difficulty changing a long-established habit or practice	*‘(GBMSM] have really in their fundamentals that you know, you need to test every three or four* months’—GBMSM Stakeholder 161	Feedback on behaviour Prompts/cues	GBMSM stakeholders	**Intervention J** Automated digital prompts could be sent to GBMSM who are identified as screening more frequently, to gently remind them of the changed guidelines.

GBMSM, gay, bisexual, and other men who have sex with men; STIs, sexually transmitted infections.

### Knowledge and trust

*Confidence and trust in the rationale behind reducing the frequency of asymptomatic screening* was a theme in participant responses. This theme contained elements of the knowledge and social influence (see the Social influence and identity section). Beliefs about the natural history of Ng and Ct may have shaped GBMSM stakeholders’ confidence in the rationale. Some participants believed all STIs require treatment to be resolved, which may have heightened anxiety about infrequent screening and contributed to feelings of neglect (see the Emotional responses and habit section).

For professional stakeholders*, priority of the goal of reducing unnecessary antibiotic use* was a theme. Sentiments within this theme varied. While some emphasised the importance of reducing AMR, were concerned that the risk was insufficiently quantified. For some, the potential introduction of doxycycline postexposure prophylaxis (doxy-PEP) was hard to reconcile with reducing asymptomatic screening for Ng and Ct to prevent AMR because of the potential for doxy-PEP to cause AMR.

### Social influences and identity

There was also a social influence element to the theme of *confidence and trust in the rationale behind reducing the frequency of asymptomatic screening*. There was mistrust of stated intentions, among some GBMSM, who suggested changes were to cut costs at the expense of GBMSM. The belief that all STIs require treatment to be resolved may have exacerbated this (see Knowledge, trust and goals section).

*Identity as a member of a GBMSM community* was also a theme among GBMSM stakeholders. GBMSM noted that infrequent screening could harm social standing, as regular STI screening was seen as an expectation in the community. Not wanting to deter potential partners was cited as a barrier to following reduced screening frequency. This was mentioned by GBMSM of different ages and backgrounds but was heard most often from participants with more sexual partners. Frequent screening was apparently an important aspect of participant’s identities as GBMSM community members, especially for GBMSM with more sexual partners.

For professional stakeholders, the contrast between the new guidance and current practice was central to the theme of *identity as a sexual health service professional*. Unease stemmed from the potential for the policy to undermine previous messaging. There was also an element of social influence here, with professional stakeholders suggesting some of their colleagues would be *very against this sort of change*.

### Context and resources

The *burden on GBMSM from screening* was a consistent theme. Frequent screening was described as an arduous process by some GBMSM and professional stakeholders. Notably, these sentiments were more common among stakeholders living or working outside London. However, GBMSM stakeholders had concerns about their ability to recognise symptoms and know when to test under new guidelines. Remaining constantly vigilant for symptoms could be burdensome itself. Some GBMSM stakeholders also noted that securing testing appointments on an ad hoc basis was potentially more difficult than scheduling regular screening appointments in advance. Thus, while reducing asymptomatic screening may lessen one burden, it could introduce new challenges that offset this.

### Concern about consequences

Concerns about consequences included: *concern about the personal sexual health impact*, *concern about the community sexual health impact*, *concerns about the structural sexual health impact* and *concerns about the impact on attitudes towards Ng, Ct and other STIs*. With respect to *concern the impact personal sexual health impact,* GBMSM stakeholders mentioned the uncertain long-term consequences of untreated STIs, particularly for those with pre-existing health conditions. Some of these concerns are better understood in the context of the belief, held by some GBMSM, that all STIs would only resolve with active treatment (see Knowledge, trust and goals section). There were also concerns that contracting an STI from an early sexual experience could carry a psychological burden that might be *detrimental to* (overall) *sexual experience*. In terms of *concern about the community sexual health impact*, GBMSM stakeholders mentioned concern about passing an STI to a partner, and concern about potential increases in the general prevalence of STIs from both groups. There was specific worry over untreated Ct and Ng in bisexual GBMSM infecting women, with professional and GBMSM stakeholders considering untreated infections among this group to be an important factor.

In relation to *concerns about the structural sexual health impact*, GBMSM and professional stakeholders viewed reduced asymptomatic screening as potentially freeing capacity for symptomatic cases, though there was concern from GBMSM stakeholders that reduced demand could lead to service cuts. There was also concern that the change could reduce contact between GBMSM and sexual health services, resulting in less opportunity for *behaviour change messaging, talking about partners and following up*. Professional stakeholders recognised that the change could introduce challenges when evaluating new interventions. Reduced screening could amount ‘*changing the goalposts’*, at the same time as potentially introducing 4CMENB vaccination and Doxy-PEP. They also suggested contracts incentivising high testing rates may require renegotiation, and they expressed doubt about who would benefit from financial savings. On the positive side, professional stakeholders saw potential for time and cost savings, possibly supporting more comprehensive sexual healthcare.

With respect to *concerns about the impact on attitudes towards Ng, Ct and other STIs*, GBMSM and professional stakeholders suggested the guideline changes could downgrade perceived STI severity. This could lower stigma for Ct and Ng, perhaps reducing the impact of social disapproval as a constraint on changes to screening practices (see the Social influence and identity section). At the same time, it was acknowledged that the same attitude change could also risk trivialising HIV and syphilis.

### Emotional responses and habit

Participants’ emotional responses centred around two key themes: *Anxiety about infrequent screening* and a *sense of neglect regarding GBMSM sexual health*. Anxiety was often rationalised by GBMSM as a response to concerns about the personal, community and structural sexual health impacts as well as the incongruence of reduced screening with their *identity as a GBMSM community member*. Feelings of neglect stemmed from a perception that cost-cutting measures overlook GBMSM health and well-being. This sentiment was exacerbated by mistrust and the belief that all STIs must be treated to be resolved (see the Knowledge, trust and goals section). The theme was particularly strong among older GBMSM stakeholders, some of whom attributed their concerns to experiences during the HIV pandemic.

Another theme, *difficulty changing a long-established habit or practice,* was apparent from the way screening was described as an established routine by GBMSM stakeholders. GBMSM stakeholders described public health messaging around frequent STI screening being *in their fundamentals*.

### Potential interventions

Mapping TDF domains to BCTs resulted in a number of potential intervention components. Candidate interventions were reviewed by the multidisciplinary team using APEASE criteria and are outlined in table 2.

## Discussion

### Main findings

The acceptability of reducing asymptomatic Ct and Ng screening frequency was low among the GBMSM we interviewed. In contrast, professional stakeholders were more accepting, possibly due to greater knowledge and the absence of personal health implications. As a marginalised group, GBMSM stakeholders may also be more sensitive to perceived neglect. Nevertheless, both groups identified barriers and facilitators within key themes: knowledge, trust and goals; concerns about consequences; social influence and identity; context and resources and emotional responses and habit.

To address knowledge, trust and goals barriers, interventions should include providing information to both professional and GBMSM stakeholders, facilitating discussions with GBMSM and community organisations to identify shared goals, and setting reduction targets for inappropriate antibiotic prescriptions among sexual health professionals.

For social influence and identity, we recommend using credible sources to communicate guidelines, providing behavioural substitutes for testing and leveraging peer support among GBMSM stakeholders.^18^

Context and resource barriers could be mitigated with guidance on how to make the best use of available screening resources. Empathy-based communication by sexual health service professionals, alongside digital prompts for those screening unnecessarily frequently, could help address emotional and habitual barriers among GBMSM.^18^

### Findings in the context of current literature

The attachment to screening we observed among GBMSM stakeholders, parallels previous research.^19^ Specifically, the perception that the guideline changes were really motivated by a desire to cut costs at their expense, has been documented in the existing literature.^19^ Indeed, these concerns can remain a barrier to screening deimplementation even when substitution behaviours are provided.^20^ Consistent with our own suggestions, previous qualitative studies have suggested that proactive communication with both groups in the form of education and outreach could mitigate some of these barriers.^20^

There are also parallels between the role of social influence in STI screening and its influence on HIV pre-exposure prophylaxis (PrEP) use. While GBMSM participants in our study experienced or anticipated stigma for attending STI screening, this was generally outweighed by social pressure from within their community. As with HIV PrEP, STI screening is both stigmatised for its perceived link to sexual promiscuity and reinforced as a social norm in many GBMSM groups.^21^,^23^ This highlights the complexity of social influences, where behaviours can be both stigmatised and socially reinforced. Any interventions leveraging social influence must account for this duality.

These findings align with broader health intervention deimplementation literature. Anxiety, social norms and perceptions of health risks are known barriers to reducing unnecessary screening.^24^ This literature also warns that reducing inappropriate screening could unintentionally lower engagement with necessary screening.^24^ Both professional and GBMSM stakeholders raised this concern, suggesting guideline changes might trivialise other STIs such as hepatitis, syphilis and HIV.

### Strengths and limitations

We developed practical suggestions from our thematic analysis findings. Mapping barriers and facilitators to BCTs to create evidence-based intervention recommendations is a key strength of this research. By translating rich qualitative data into actionable insights, we provide policymakers with a clear path forward in a complex public health landscape.

While this study offers valuable insights, its limitations must be acknowledged. The sample was diverse in age, sex assigned at birth, ethnicity, region and sexual health behaviours. However, recruitment targeted individuals highly engaged with sexual health services. Although this group is most affected by guideline changes, policymakers should also consider the needs of less engaged GBMSM.

### Implications for policy and practice

If national guidelines move towards reducing asymptomatic Ct and Ng screening for GBMSM, our recommendations could support deimplementation. This should include further stakeholder workshops to codesign culturally sensitive, acceptable and inclusive interventions, using implementation science tools where appropriate (eg, normalisation process theory^25^).

Both stakeholder groups raised concerns about the potential of a higher risk of transmission from behaviourally bisexual men to female partners if screening frequency was reduced among GBMSM. Proponents of reduced asymptomatic Ng and Ct screening in GBMSM have suggested that specific guidelines for bisexual men may be needed.^3^ However, the acceptability of this approach is unknown, and distinguishing between homosexual and bisexual men could have unintended stigmatising effects. Policymakers should consider whether there is epidemiological evidence to suggest that reducing screening frequency among GBMSM would lead to an increased risk of infection and associated harms among female partners of bisexual men. Any change in policy should consider unintended consequences including the potential for reinforcing biphobic discourse. Additionally, as bisexual men engage with sexual health services less than men who have sex exclusively with men,^26^ any guideline changes must avoid exacerbating existing health inequities.

Two novel interventions that could impact the acceptability of deimplementing asymptomatic Ng and Ct screening are bacterial STI PEP with doxycycline and the 4CMENB vaccine for gonorrhoea prevention. Although neither is currently available for STI prevention in the UK, the Joint Committee on Vaccination and Immunisation has recommended 4CMENB, and studies indicate that 8%–9% of HIV PrEP users have already used antibiotic STI PEP.^27 28^ Future research should explicitly examine the interdependencies between emerging interventions and the deimplementation of Ng and Ct screening in GBMSM.

## Conclusions

This study highlights the considerable challenges that would face any attempt to reduce asymptomatic Ng and Ct screening. These include knowledge gaps, negative beliefs about consequences, social influences, negative emotional responses to the new guidelines and the challenge of breaking established habits. A number of potential interventions could improve acceptance of any such guideline change and stakeholder engagement should be utilised to refine these.

## Supplementary material

10.1136/sextrans-2025-056556 online supplemental file 1

## Data Availability

Data are available upon reasonable request.
