# Supplementary material for: ‘It does fill me with a bit of unease’: a qualitative study of the acceptability, facilitators and barriers to reducing the frequency of screening for asymptomatic sexually transmitted infections among gay, bisexual and other men who have sex with men
Source: Sex Transm Infect. 2025 Jul 15;102(1):e056556. doi: 10.1136/sextrans-2025-056556 (PMC12911560; doi:10.1136/sextrans-2025-056556)
Supplement: online supplemental file 1 [file sextrans-102-1-s001.docx]

**Appendix**

**Appendix 1: GBMSM stakeholder interview topic guide**

Introduction (5 mins):

1. Confirm that the interviewee can converse safely and privately
2. Check they are happy for the conversation to be recorded and start recording
3. Interviewer to introduce themselves and explain:
   1. We will talk about sexual health today, in particular frequency of testing for some STIs
   2. That they are neutral, have no agenda and just want to hear the participant’s honest opinions.
   3. Explain that they can withdraw from the interview at any time, and that they can have their answers withdrawn in the next four months
   4. Everything will be anonymous- we may use quotes, but these will not be attributed to you
   5. The interview will take approximately one hour
   6. Reiterate that they can pause or stop the interview at any time
4. Go through information sheet and consent form (if not already done)
5. Do you have any questions?
6. Would you like a copy of the findings of the research?
7. Would you like the £30 voucher?

Personal background (5 mins) :

1. First, I’m going to ask you some background questions about yourself. This helps us to understand your life and experiences a bit more. There are no wrong answers to any of these questions, we just want to understand more about you. Please also remember that your answers will be anonymous
2. Where do you live? Urban or rural area.
3. Who do you live with?
4. What do you do for work?
5. Are you in a relationship? If so, probe for what kind (i.e. monogamous, multiple partners, casual/established etc.) Are you having sex with men or women?
6. What methods, if any, do you use to prevent STIs? Probe for PrEP

Current STI testing behaviour (10 mins):

1. In this section, we’re going to talk about your sexual health.
2. I’d like you to think about the last time you tested for any STIs (e.g., HIV, syphilis, chlamydia, gonorrhoea, hepatitis). By this we mean having a test, not testing positive.
3. When was this?
4. Where did you test?
5. What prompted you to test at this time?
6. What did you test for? Probe to understand if it was bacterial STIs only or also included HIV/ syphilis/ hepatitis through blood tests
7. How was the process of booking the tests? Was it easy/difficult? Did you need any help?
8. How did you feel emotionally during the tests?
9. Was there anything positive or negative that stood out for you? E.g. waiting times, how they were treated at the clinic
10. Before the tests we just talked about, when was the last time you tested for any STI?
11. Over the last year, how often have you tested for STIs?
12. Has this changed in the last 5 years, for example during COVID?
13. It seems like you test approximately every [fill in based on answers above] is that correct?
14. What made you choose to test at this frequency? No right or wrong answer, want to understand your decision-making process
15. You said that the last time you tested it was because [fill in from above]. Are there any other things that prompt you to test for STIs? Probe to understand relationship circumstances, life events, influence of peers, influence of clinicians, sense of “duty” etc.
16. Are you aware of any current guidelines on how often you should test for STIs? If not, explain recommendation to test every 3 months for GBMSM (see slide for details). If so: where did you learn about this recommendation?
17. If testing every 3 months: what prompts you to test every 3 months? If not testing every 3 months: what are the factors which inform your decision to test less frequently?

Reaction to changes in testing guidelines (25 mins):

1. Now we’re going to talk about a scenario in which GBMSM have been asked to stop regularly testing (i.e., every 3 months) for chlamydia and gonorrhoea, and only test if they have symptoms of these infections. They would still be recommended to test for HIV (unless they are living with HIV) and syphilis every 3 months. As many chlamydia and gonorrhoea in GBMSM are asymptomatic (especially in the throat and rectum) but are still treated with antibiotics, there are concerns that this practice could lead to antimicrobial resistance. Treating infections that will not lead to harm could also place an undue burden on GBMSM, clinicians and the healthcare system. However, this could lead to an increase in chlamydia and gonorrhoea infections among GBMSM.
2. What are your first reactions to this idea? Reiterate that we are neutral and just want to understand their point of view Is what is being considered clear to you? Do you have any questions?
3. We hope we have explained it clearly, do you have any questions?
4. How much of a change is this compared to what you are already doing? Probe to understand: will they need to break an existing habit of testing every 3 months, or form a new habit of only testing when they have symptoms?
5. If this change in guidelines was introduced, how might it influence your STI testing habits? Would you do anything differently?
6. How do you feel about this proposed change? Probe to understand any positive or negative emotions, e.g. fear/ worry over not detecting and infection; relief/ happiness over not needing to test as often; no emotional reaction
7. If you were to start testing in this way, what are your view on the positive or negative impact on: your own health b) the health of the wider GBMSM community and c) women and the wider population
8. Given the potential advantages/ disadvantages we have talked about, do you have a view on whether this is a good or bad idea?
9. Would your attitude to STIs change at all? Would your view of how important it is to test for STIs change? Probe on if perception of seriousness of STIs has changed
10. Would your sexual behaviour change at all? Could explain further as: more consideration for condoms
11. If you were recommended to test in this way, would you follow these guidelines?
12. How would you decide to have a test for chlamydia or gonorrhoea if there is not a set recommended interval?
13. Would you test with your own preferences and needs, regardless of what the guidelines state?
14. To understand: would they pay for a test? Would they say they had symptoms if they didn’t?
15. If testing guidelines changed, how do you think the people around you would react?
    1. Your partner(s)
    2. Your friends (generally, and those who are GBMSM)
    3. Your family
16. To what extent, if at all, would their reaction change how you approach STI testing?

****Ask if the participant is ok to continue or if they need a short break *****

I’d like to discuss how this change could work in practice:

1. Can mention to participant this section may feel repetitive
2. If you were asked to test every 6 months, even if you did not have any symptoms, how different would this be compared to what you currently do?
   1. What are the best/ worst things that could happen because of this change?
3. If you were asked to test every 12 months, even if you did not have symptoms, how different would this be compared to what you currently do?
   1. What are the best/ worst things that could happen because of this change?
4. If taking PrEP: how often do you need to have STIs tests as part of your PrEP monitoring?
   1. How would you feel if chlamydia/gonorrhoea testing was no longer part of these visits?
5. Imagine now that a vaccine that is partially effective in protecting you against gonorrhoea is available. Please assume that this vaccine is free and available at sexual health clinics. It is not available now, so this is just a hypothetical situation
   1. If you had access to this vaccine, might this change if/ how often you would test?
   2. Extra information about Bexsero if needed: This vaccination was originally used to prevent bacterial meningitis in children. Studies suggest that two doses of Bexsero reduces the chances of getting gonorrhoea by approximately 40% (individual studies have said 33%, 40% and 46%). The Hepatitis A and B vaccines are about 90% effective
6. Imagine now that a pill/ antibiotic is available that you take after sex that reduces the chances that you will get a bacterial STI such as chlamydia or syphilis (this is sometimes called dPEP, doxyPEP or STI PEP). Please assume that this pill is free and available at sexual health clinics. It is not available now, so this is just a hypothetical situation
   1. If you had access to this pill, might this change if/ how often you would test?
   2. Extra information about dPEP if needed: STI prophylaxis involves taking an antibiotic pill to prevent bacterial STIs, such as syphilis and chlamydia. So far, research has focused on taking two doses soon after sex. The antibiotic seems to prevent bacterial growth and makes it less likely for exposure to lead to infection. Three studies have shown the effective of using an antibiotic called doxycycline preventatively.

Communication strategies (10 mins):

1. In this section, we’re going to talk about how you would prefer to hear information about this new recommendation for testing frequency.
2. What was the last piece of information about sexual health that you remember seeing? Prompt to recollect e.g. any posters/ advertising campaigns, advice from a clinician, messages on social media
   1. What was the message it was trying to convey?
   2. Where did you see it/ who did you hear it from?
   3. What did you like/ not like about it?
   4. Did you do anything because of receiving this information? Why/why not?
3. If the guidance changed and you were no longer asked to test for certain STIs every 3 months (but still were recommended to test for syphilis and HIV every 3 months):
   1. What information would you need to have? E.g. reasons why this has been done, what it means for your sexual health
   2. Who would you trust to give you this information? Probe to understand how they would react if it came from SHS staff
   3. What format would be best to give this information?
   4. What could support the GBMSM community as a whole in this change?
   5. Is there anything else you think could be done to help support you if the current guidelines were changed?

Closing (5 mins):

1. Is there anything else you would like to say that you haven’t had the chance to yet?
2. Do you have any questions?
3. Did you find talking in the discussion helpful?
4. Sometimes this can be a sensitive issue - did you find the discussion comfortable?
5. Thank and close

**Appendix 2: Professional stakeholder interview topic guide**

Introduction (5 mins):

1. Confirm that the interviewee can converse safely and privately
2. Check they are happy for the conversation to be recorded and start recording
3. Interviewer to introduce themselves and explain:
   1. We will talk about sexual health today, in particular frequency of testing for some STIs
   2. That they are neutral, have no agenda and just want to hear the participant’s honest opinions.
   3. Explain that they can withdraw from the interview at any time, and that they can have their answers withdrawn in the next four months
   4. Everything will be anonymous- we may use quotes, but these will not be attributed to you
   5. The interview will take approximately one hour
   6. Reiterate that they can pause or stop the interview at any time
4. Go through information sheet and consent form (if not already done)
5. Do you have any questions?
6. Would you like a copy of the findings of the research?
7. Would you like the £30 voucher?

Professional background (5 mins) :

1. First of all, I’m going to ask you some background questions about yourself. This helps us to contextualise your answers when we analyse our data
2. Where do you currently work?
3. What is the goal/ remit of your organisation?
4. How would you describe your role? (Understand: population groups they work with, topics they engage with, geographic scope)
5. To what extent are you involved with encouraging or promoting STI testing among GBMSM?

Reactions to testing scenario (35 mins):

1. Now we’re going to talk about a scenario in which GBMSM have been asked to stop regularly testing (i.e., every 3 months) for chlamydia and gonorrhoea, and only test if they have symptoms of these infections. They would still be recommended to test for HIV (unless they are living with HIV) and syphilis every 3 months. As many chlamydia and gonorrhoea in GBMSM are asymptomatic (especially in the throat and rectum) but are still treated with antibiotics, there are concerns that this practice could lead to antimicrobial resistance. Treating infections that will not lead to harm could also place an undue burden on GBMSM, clinicians and the healthcare system. However, this could lead to an increase in chlamydia and gonorrhoea infections among GBMSM.
2. What are your first reactions to this idea?
3. Do you understand the rationale for this change and why it has been proposed?
4. What do you think about this way to reduce the chances of AMR?
5. Do you feel that there is enough evidence to support this recommendation?
6. How much of a change is this compared to what you already recommend/ what you already promote to your service users?
7. How do you feel about this proposed change? Probe to understand any positive or negative emotions, e.g. fear/ worry of not detecting an infection; relief/happiness over a lower burden on services
8. How easy or difficult do you think it would be to start testing in this way?
9. What, if anything, would be easy about implementing these changes?
10. What problems do you think there could be?
11. How could these problems be addressed?
12. What preparation steps would be needed before a change such as this? E.g. within your organisation
13. What, if any, would the impact of these changes be on clinic resources, such as staff or appointment availability?
14. If these guidelines were adopted, what do you think the consequences would be for your professional practice/ working life?
    1. What would be the best thing that would happen?
    2. What would be the worst thing that would happen?
    3. Do the benefits outweigh the costs?
15. If these guidelines were adopted, what do you think the consequences would be for the health of the GBMSM community:
    1. What would be the best thing that would happen?
    2. What would be the worst thing that would happen?
    3. Do the benefits outweigh the costs? Probe on AMR if not mentioned
    4. Would there be any unintended consequences?
16. Would the consequences be different for different groups? (Probe if not mentioned: PrEP users, inclusion health groups) :
17. How do you think the people you work with would react if you started to test for STIs in this way?
18. If these guidelines were adopted, what do you think the consequences would be for your colleagues within your organisation?
19. If these guidelines were adopted, what do you think the consequences would be for your colleagues outside your organisation?
20. If these guidelines were adopted, what do you think the consequences would be for your patients/ service users?
21. Whose reaction would be most important to you?
22. To what extent, if at all, would this recommendation create any tension with any of your professional responsibilities?

If GBMSM were asked to test every 6 months, even if they did not have any symptoms:

1. What do you see as pros for: you? Your organisation? Service users?
2. What do you see as cons for: you? Your organisation? Service users?

If GBMSM were asked to test every 12 months, even if they did not have symptoms:

1. What do you see as pros for: you? Your organisation? Service users?
2. What do you see as cons for: you? Your organisation? Service users?

For GBMSM taking PrEP and who may expect STI tests as part of their monitoring visits:

1. What do you see as pros for: you? Your organisation? Service users?
2. What do you see as cons for: you? Your organisation? Service users?

If the guidelines changed to include a recommendation that GBMSM at high risk of an STI should take doxycycline prophylaxis (referred to as STI PEP, dPEP or doxyPEP) to prevent infection:

1. What do you see as pros for: you? Your organisation? Service users?
2. What do you see as cons for: you? Your organisation? Service users?

If a vaccine (e.g. BexSero) were available that was partially effective in protecting GBMSM from gonorrhoea:

1. What do you see as pros for: you? Your organisation? Service users?
2. What do you see as cons for: you? Your organisation? Service users?

Communication of the change (10 mins):

1. If the recommendation that GBMSM should test for certain STIS every 3 months were removed, how do you see your (or your organisation’s) role in communicating this to service users?
2. How would you communicate this change with your patients/ service users?
3. What support or information would you need to do so effectively?
4. How would you react to/ deal with potential resistance from patients or service users?
5. Which other groups would see as important in spreading/ consolidating the message of a change in guidance?
6. Which other strategies do you think would be needed to support a change such as this?

Closing (5 mins):

1. Is there anything else you would like to say that you haven’t had the chance to yet?
2. Do you have any questions?
3. Thank and close
